# Physical activity and long-term fatigue among colorectal cancer survivors – a population-based prospective study

**DOI:** 10.1186/s12885-020-06918-x

**Published:** 2020-05-18

**Authors:** Ruth Elisa Eyl, Melissa S. Y. Thong, Prudence R. Carr, Lina Jansen, Lena Koch-Gallenkamp, Michael Hoffmeister, Jenny Chang-Claude, Hermann Brenner, Volker Arndt

**Affiliations:** 1 Division of Clinical Epidemiology and Aging Research, German Cancer Research Center (DKFZ), Im Neuenheimer Feld 581, 69120 Heidelberg, Germany; 2 Unit of Cancer Survivorship, Division of Clinical Epidemiology and Aging Research, German Cancer Research Center (DKFZ), Im Neuenheimer Feld 581, 69120 Heidelberg, Germany; 3 Unit of Genetic Epidemiology, Division of Cancer Epidemiology, German Cancer Research Center (DKFZ), Im Neuenheimer Feld 581, 69120 Heidelberg, Germany; 4 Cancer Epidemiology Group, University Cancer Center Hamburg (UCCH), University Medical Center Hamburg-Eppendorf, Martinistraße 54, 20251 Hamburg, Germany; 5 Division of Preventive Oncology, German Cancer Research Center (DKFZ) and National Center for Tumor Diseases (NCT), Im Neuenheimer Feld 280, 69120 Heidelberg, Germany; 6 German Cancer Consortium (DKTK), German Cancer Research Center (DKFZ), Im Neuenheimer Feld 280, 69120 Heidelberg, Germany

**Keywords:** Physical activity, Fatigue, Colorectal cancer, Long-term survivorship

## Abstract

**Background:**

Evidence suggests that physical activity (PA) is beneficial for reducing fatigue in colorectal cancer (CRC) survivors. However, little is known regarding long-term effects of PA on fatigue and whether pre-diagnosis PA is associated with less fatigue in the years after diagnosis. Our study aimed to investigate the association of pre- and post-diagnosis PA with long-term fatigue in CRC survivors.

**Methods:**

This study used a German population-based cohort of 1781 individuals, diagnosed with CRC in 2003–2014, and alive at five-year follow-up (5YFU). Physical activity was assessed at diagnosis and at 5YFU. Fatigue was assessed by the Fatigue Assessment Questionnaire and the EORTC Quality of Life Questionnaire-Core 30 fatigue subscale at 5YFU. Multivariable linear regression was used to explore associations between pre- and post-diagnosis PA and fatigue at 5YFU.

**Results:**

No evidence was found that pre-diagnosis PA was associated with less fatigue in long-term CRC survivors. Pre-diagnosis work-related PA and vigorous PA were even associated with higher levels of physical (Beta (ß) = 2.52, 95% confidence interval (CI) = 1.14–3.90; ß = 2.03, CI = 0.65–3.41), cognitive (ß = 0.17, CI = 0.05–0.28; ß = 0.13, CI = 0.01–0.25), and affective fatigue (ß = 0.26, CI = 0.07–0.46; ß = 0.21, CI = 0.02–0.40). In cross-sectional analyses, post-diagnosis PA was strongly associated with lower fatigue on all scales.

**Conclusions:**

In this study, pre-diagnosis PA does not appear to be associated with less fatigue among long-term CRC survivors. Our results support the importance of ongoing PA in long-term CRC survivors. Our findings might be used as a basis for further research on specific PA interventions to improve the long-term outcome of CRC survivors.

## Background

With over 1.8 million estimated incident cases and 881,000 estimated deaths in 2018, colorectal cancer (CRC) is the third most common cancer and the second most common cause of cancer-related death worldwide [1]. Early detection and improvements in treatment as well as the aging of the population have substantially contributed to the increasing number of CRC survivors [2, 3]. In developed countries, CRC survivors represent the third largest cancer survivor group next to breast and prostate cancer survivors [4].

Many CRC survivors still experience detriments in (health-related) quality of life (QOL) years after their diagnosis [5–7] and fatigue has been reported to affect QOL more than other symptoms such as pain or depression [8, 9]. Therefore, it is of great relevance to identify interventions that have the potential to decrease fatigue in CRC survivors and thereby improve the QOL of this population.

Physical inactivity is an important modifiable risk factor for non-communicable diseases including CRC [10]. Furthermore, evidence has accumulated that physical activity (PA), especially leisure time PA is prognostically relevant for CRC patients. Aside from a better prognosis for CRC survivors who are physically active [11–14], studies reported that CRC survivors who were more physically active tended to report less fatigue [15–18].

Although one study [19] investigated the association of pre-diagnosis PA and fatigue 2 years after diagnosis so far, no study has investigated associations of pre- as well as post-diagnosis PA with fatigue specifically in long-term (≥5 years post-diagnosis) CRC survivors. Moreover, the available evidence regarding the association between PA and fatigue among CRC survivors is mainly based on studies with a cross-sectional design [15–18].

Recent studies assessing PA after treatment [16, 20–22] and also prehabilitation programs including PA before cancer treatment [23–25] found PA to be beneficial for cancer survivors’ physical and psychological health. Furthermore, it has been reported that exercise/PA might have long-lasting effects on individuals’ health [26–28]. Therefore, we hypothesized that pre-diagnosis PA might be beneficial for the fatigue of long-term CRC survivors since survivors who were physically active before diagnosis may already have laid a basis of positive lifestyle strategies that they may use to maintain well-being during treatment and in the years of survivorship. The aim of this study was therefore to additionally investigate the prospective association between pre-diagnosis PA and fatigue in long-term CRC survivors. Further, this study investigated the potential effects of different domains of pre-diagnosis PA such as leisure time and work-related PA as well as different PA intensities on fatigue of long-term CRC survivors.

## Methods

### Study design

This analysis is based on CRC patients recruited within the ongoing population-based DACHS (Darmkrebs: Chancen der Verhütung durch Screening) study. The study is carried out in the Rhine-Neckar region in the southwest of Germany; an area that has a population of about 2 million people. To date, the study includes over 6000 patients with both symptomatic and screen-detected CRC, recruited since 2003. Eligible cases with a histologically confirmed diagnosis of primary CRC (International Classification of Diseases, 10th Revision [ICD-10] codes C18-C20) have to be older than 30 years at diagnosis, residents of the study region, German speaking, and physically and mentally able to participate in an interview of approximately 1 h. Approximately 50% of all eligible patients are recruited by 22 hospitals in the study area. Incomplete recruitment of patients is largely due to lack of time among the clinicians in charge of notifying the study center in the routine setting. Further details of the study have been described elsewhere [11, 29–31]. The DACHS study was approved by the ethics committees of the University of Heidelberg and the state medical boards of Baden-Wuerttemberg and Rhineland-Palatinate. All participants gave written informed consent.

### Data collection and follow-up

Patients with newly diagnosed CRC are identified by their treating clinician during their hospital stay and are interviewed in the hospital or contacted by mail shortly after their discharge by clinicians or clinical cancer registries. At baseline, sociodemographic information, medical, and lifestyle history (including PA) are obtained by trained interviewers using a standardized questionnaire. Three years after diagnosis, detailed information about treatment, other diseases, and recurrence is collected from attending physicians, using a standardized questionnaire. In order to obtain follow-up data including changes in lifestyle (including PA), medical, or recurrence history, and fatigue, CRC patients are sent a questionnaire by mail 5 years after diagnosis. Information about recurrence, other diseases, and new cancers is verified by the patients’ physicians. Patients’ vital status is regularly checked through population registries.

### Study population

**Fig. 1:**
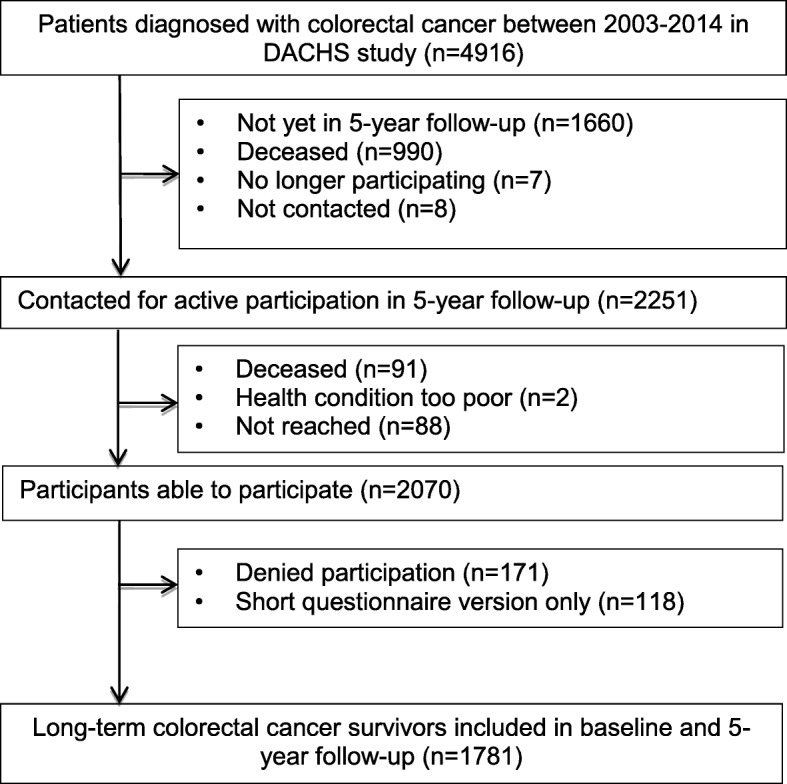
Flow diagram of patients with colorectal cancer included in the analyses

For this analysis, 1781 participants who were recruited between 2003 and 2010 and participated in the five-year follow-up (5YFU) between 2009 and 2016 were included (see Fig. 1 for detailed information on participants included in the analysis).

### Assessment of physical activity

At baseline, information on retrospective PA was collected by trained interviewers in a personal interview for each age decade between 20 and 80 years, depending on participant’s age at diagnosis. Patients were asked for the hours per week they had engaged in different activities. One question was asked to estimate the amount of time spent on hard work-related PA (e.g. in agriculture, as health care worker or in the military), one question on light work-related PA (housework, gardening, as sales person, hairdresser), one question on walking (e.g. going for walks, going shopping, walking to and/or home from work), one question on cycling (e.g. means of transportation in everyday life, using the bike to and/or home from work), and one question on sports (e.g. soccer, swimming, skiing, mountain climbing, jogging). These retrospective data have been used to address the prognostic impact of PA in recent papers [11, 32]. Five years after CRC diagnosis, information on average PA during the past week was assessed with a mailed questionnaire that included the short-form of the International Physical Activity Questionnaire (IPAQ). The questionnaire asks for the number of days and minutes per week spent with vigorous PA e.g. jogging, moderate PA e.g. swimming, walking, and sitting.

Based on activity-specific metabolic equivalent (MET) score values described by Craig et al. [33], MET hours per week (MET-h/wk) were calculated according to activities performed at baseline and at 5YFU. The following task-specific MET-h/wk score values were used at baseline: hard work = 8 MET-h/wk, light work = 2.5 MET-h/wk, walking = 3.3 MET-h/wk, cycling = 6 MET-h/wk, sports = 8 MET-h/wk; and at 5YFU: vigorous PA = 8 MET-h/wk, moderate PA = 4 MET-h/wk, and moderate walking = 3.3 MET-h/wk While from both assessment methods these MET-h/wk can be derived, the wider range of PA domains assessed at baseline compared to the 5YFU and the difference in the assessment methods (personal interview and mail) might hamper the comparability of the obtained METs from baseline and 5YFU and should be kept in mind.

From the baseline assessment, activity-specific lifetime MET-h/wk were derived from the MET-h/wk spent at ages 20, 30, 40, 50, 60, 70, and 80 (assessed at baseline), considering the current age at diagnosis of the patient and the years spent in each decade. Information from the age decade preceding the patients’ current age at diagnosis was used to calculate the activity-specific MET-h/wk for the last age decade (e.g. PA at diagnosis age 60 for participants in the age group 60–69). The activity-specific MET-h/wk were summed up to create the variables baseline PA lifetime and last decade.

In subgroup analyses, baseline PA was categorized into different PA domains (leisure time PA [walking, cycling, sports] and work-related PA [light work, hard work]) and intensities (light PA [light work], moderate PA [walking], and vigorous PA [cycling, sports, hard work]). Physical activity was classified according to the second version of the Physical Activity Guidelines for Americans [34]: light-intensity PA = 1.1–2.9 METs, moderate PA = 3–5.9 METs, and vigorous PA = ≥6 METs.

From the 5YFU, the MET-h/wk of the last week were calculated for each of the specific activity types and then summed up to obtain the 5YFU PA.

Based on sample distribution, quartiles (Q) for PA at baseline for the last age decade (Q1 = < 74.7 MET-h/wk, Q2 74.7- < 118.3 MET-h/wk; Q3 118.3- < 183.0 MET-h/wk; ≥183.0 MET-h/wk) and 5YFU (Q1 = < 11.6, Q2 = 11.6- < 34.1, Q3 = 34.1- < 79.0, Q4 = ≥79.0) were calculated. Patients in Q1 were defined as physically inactive whereas patients in Q2-Q4 were defined as physically active. To assess associations of different PA levels with fatigue, the lowest quartile was used as the reference category. Further, these quartiles were used to classify survivors in four groups: active maintainers (active at baseline and at 5YFU), increasers (inactive at baseline, active at 5YFU), decreasers (active at baseline, inactive at 5YFU), and inactive maintainers (inactive at baseline and at 5YFU).

For the main analyses, baseline PA information of the last decade was used and defined as pre-diagnosis PA whereas PA at 5YFU was defined as post-diagnosis PA.

### Assessment of fatigue

At 5YFU, fatigue was measured using the Fatigue Assessment Questionnaire (FAQ) developed by Glaus et al. [35], and the Quality of Life Questionnaire-Core 30 (QLQ-C30) [36] which was developed by the European Organization for Research and Treatment of Cancer (EORTC). The FAQ assesses the dimensions physical, cognitive, and affective fatigue. Since in the DACHS study, only the cognitive (3 items) and affective (5 items) questions of the FAQ were assessed, the fatigue scale of the QLQ-C30 (3 items) was included to additionally assess the physical aspect of fatigue [37, 38]. Scoring was performed according to the FAQ and the QLQ-C30 scoring manuals [35, 39]. Cognitive scores were linearly transformed to a 0–9 point scale, affective scores to a 0–15 point scale, and physical fatigue to a 0–100 point scale. Lower scores on cognitive, affective, and physical fatigue imply less fatigue.

### Statistical analysis

To estimate the ordinal association between pre- and post-diagnosis PA, Kendall rank correlations were calculated. Adjusted means were computed using multivariable linear regression models to explore the association of pre-diagnosis PA quartiles with fatigue. Comprehensive covariate adjustment included baseline variables such as age, sex, marital status, residential area, education, comorbidities, alcohol intake, smoking, body mass index (BMI), cancer site, cancer stage, radiotherapy, chemotherapy, and stoma.

Multivariable linear regression analyses were repeated, calculating beta values (ß) with 95% confidence intervals (CI) and modeling pre-diagnosis PA as a continuous variable (per 100 MET-h/wk) for different domains (leisure time vs. work-related) and intensities of PA (low vs. moderate vs. vigorous) with fatigue. In order to assess the independent association of the PA domains with fatigue, the multivariable models were additionally mutually adjusted for the other domain. The same procedure was implemented for the intensities of PA.

Additionally, multivariable linear regression models were calculated to explore the association between post-diagnosis PA quartiles and fatigue. Covariate adjustment included the same covariates (updated with information at 5YFU) as used in the analysis of pre-diagnosis PA and fatigue. In sensitivity analyses, pre-diagnosis PA was added to the model, and in a second step CRC recurrence. Since the results did not substantially change using the additional covariate adjustments, only results of the first covariate adjustment are reported. Moreover, partial r^2^-values were calculated to assess the independent proportion of the explained variance of fatigue by pre- and post-diagnosis PA after adjustment for potential confounders.

Multiple linear regression models were repeated for the association between changes in PA and fatigue, using the same covariates (updated with information at 5YFU) as used in the analysis of pre-diagnosis PA and fatigue.

Complete case analyses were performed since the proportion of missing values was generally low. Information regarding fatigue at 5YFU was missing in less than 2.5% of all cases. No adjustment for multiple testing was performed, given the exploratory nature of the analysis. The statistical software SAS 9.4 (SAS Institute) was used to perform all data analyses. All statistically significant results mentioned in this study refer to a *p*-value < 0.05 in two-sided testing.

## Results

Overall, 1781 long-term CRC survivors were included in the analysis. Participants were on average 66.1 years old at baseline and 60% were male and 40% female (Table 1). The tumor was located in the colon in almost 60% of participants, and confined to the intestine (UICC stage I or II) in around 60% of all cases. Primary treatment included radiotherapy and chemotherapy in 20 and 42% of cases, respectively. Five years after diagnosis, 23% of all survivors still had a stoma and around 9% of the survivors had experienced a CRC recurrence. Average pre-diagnosis PA levels were two to three times higher than post-diagnosis PA levels. The comparison of pre- and post-diagnosis PA quartiles revealed a weak correlation (Kendall rank correlation coefficient: pre-diagnosis PA, last decade = 0.16; *p* < 0.0001; pre-diagnosis PA, lifetime = 0.07; *p* < 0.0001). The correlation between pre-diagnosis PA of the last decade and the lifetime pre-diagnosis PA was stronger (Kendall rank correlation = 0.37).

**Table 1:** Colorectal cancer participant characteristics

	Total sample		Total sample
	**N (Col. %)**		**N (Col. %)**
**Overall**	1781	**Overall**	1781
Age		Pre-diagnosis PA^a^ (MET-h/wk)	
30–59 years	431 (24.2)	Q1 (< 74.7)	440 (25.0)
60–69 years	655 (36.8)	Q2 (74.7- < 118.3)	438 (24.9)
70–79 years	560 (31.4)	Q3 (118.3- < 183.0)	439 (24.9)
80+ years	135 (7.6)	Q4 (≥183.0)	443 (25.2)
Mean (SD)	66.1 (9.9)	Mean (SD)	143.5 (107.1)
Sex		Post-diagnosis PA^b^ (MET-h/wk)	
Female	706 (39.6)	Q1 (< 11.6)	447 (25.5)
Male	1075 (60.4)	Q2 (11.6- < 34.1)	428 (24.4)
Marital status^c^		Q3 (34.1- < 79.0)	441 (25.1)
Unmarried	89 (5.0)	Q4 (≥79.0)	438 (25.0)
Married	1338 (75.1)	Mean (SD)	54.9 (60.4)
Divorced	106 (6.0)	Cancer site^c^	
Widowed	245 (13.8)	Proximal colon	524 (29.4)
Residential area		Distal colon	510 (28.6)
Village (< 10,000)	635 (35.7)	Rectum	742 (41.7)
Small town	611 (34.3)	Cancer stage^c^	
City (> 100,000)	535 (30.0)	I	511 (28.7)
Education^c^		II	616 (34.6)
≤ 9 years	1153 (64.7)	III	591 (33.2)
10–11 years	312 (17.5)	IV	56 (3.1)
≥ 12 years	313 (17.6)	Detection of cancer	
BMI (kg/m^2^)^c^		Symptoms	1165 (65.4)
< 25	651 (36.6)	Screening	517 (29.0)
25- < 30	781 (43.9)	Other	99 (5.6)
≥ 30	347 (19.5)	Radiotherapy^c^	
Smoking^c^		Yes	353 (19.8)
Never	763 (43.0)	No	1427 (80.1)
Former (> 1 year)	760 (42.8)	Chemotherapy^c^	
Current	253 (14.3)	Yes	751 (42.2)
Alcohol (grams/day)^d^		No	1029 (57.8)
None	456 (25.6)	Stoma^e^ at 5YFU	
0.9–6.1	360 (20.2)	Yes	405 (22.7)
> 6.1–14.4	292 (16.4)	No	1327 (74.5)
> 14.4–30.7	330 (18.5)	Recurrence^c^ at 5YFU	
> 30.7	319 (17.9)	Yes	162 (9.1)
Comorbidities^c,g^		No	1617 (90.8)
< 2	945 (53.1)		
≥2	835 (46.9)		

^a^last age decade before diagnosis; ^b^at 5-year follow-up; ^c^1–10 missings; ^d^11–27 missings; ^e^47 missings; ^f^linear model age-adjusted; ^g^including heart attack, heart failure, stroke, diabetes, depression, other cancers, hypotension, circulatory disturbances heart, circulatory disturbances brain, circulatory disturbances legs, gout, arthritis, rheumatism, arthrosis, morbus crohn, colitis ulcerosa; Abbreviations: *Col.* column, *SD* Standard deviation, *BMI* Body mass index, *PA* Physical activity, *MET-h/wk* Metabolic equivalent hours per week, *5YFU* 5-year follow-up; apart from post-diagnosis PA, stoma, and recurrence all presented variables only include baseline information

### Association of pre- and post-diagnosis physical activity with fatigue

**Fig. 2:**
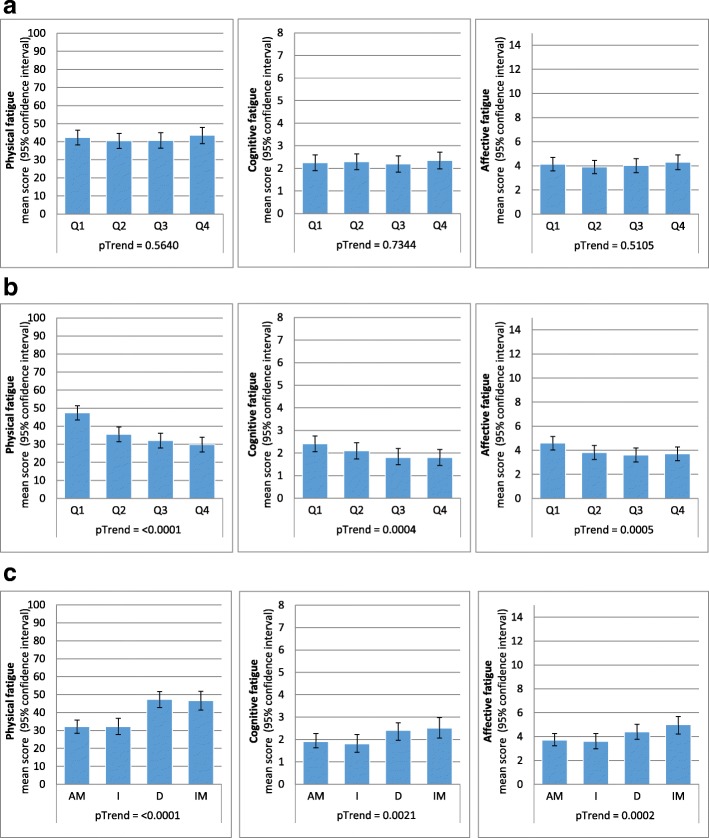
Associations between pre-, post-diagnosis and changes in physical activity and fatigue. **a**: Associations between pre-diagnosis (last decade) physical activity and fatigue. **b**: Associations between post-diagnosis physical activity and fatigue. **c**: Associations between changes in physical activity from pre- to post-diagnosis and fatigue. Abbreviations: *Q* physical activity quartile (Q1 = inactive, Q2-Q4 = active), *AM* active maintainers, *I* increasers, *D* decreasers, *IM* inactive maintainers,*5YFU* five-year follow-up, *BMI* body mass index. Footnote: Linear regression analyses adjusted for **a**: age at baseline, sex, marital status, residential area, education, number of comorbidities at baseline, alcohol intake at baseline, smoking at baseline, BMI at baseline, cancer site, cancer stage, treatment, stoma; **b** and **c**: age at 5YFU, sex, marital status, residential area, education, number of comorbidities including information from baseline until 5YFU, alcohol intake at 5YFU, smoking including information from baseline until 5YFU, BMI at 5YFU, cancer site, cancer stage, treatment, stoma

As shown in Fig. 2a, survivors who were physically active pre-diagnosis did not report significantly lower physical, cognitive, or affective fatigue 5 years post-diagnosis compared to survivors who were physically inactive pre-diagnosis. Pre-diagnosis PA also explained very little of the variance of long-term fatigue with 0.2% on the physical, 0.06% on the cognitive fatigue, and 0.1% on the affective fatigue scale.

In cross-sectional analyses, a strong and significant association between higher post-diagnosis PA and lower physical, cognitive, and affective fatigue was found (Fig. 2b). Only the association between Q2 vs. Q1 was not significantly associated with lower cognitive fatigue. Post-diagnosis PA explained around 30% of the variability of physical fatigue but only approximately 1% of the variability of cognitive and affective fatigue. Still, a significant trend was observed for post-diagnosis PA and all fatigue scales.

In sensitivity analyses using lifetime PA instead of PA of the last decade to investigate the association between pre-diagnosis PA and fatigue, the aforementioned pattern of the results did not change (Supplementary Table 1).

### Associations between changes in physical activity from pre- to post-diagnosis and fatigue

Active maintainers and increasers scored significantly lower on all fatigue scales compared to inactive maintainers with the strongest associations for physical fatigue (Fig. 2c). No differences were found when comparing decreasers to inactive maintainers.

### Associations between different domains/ intensities of pre-diagnosis physical activity and fatigue

**Fig. 3:**
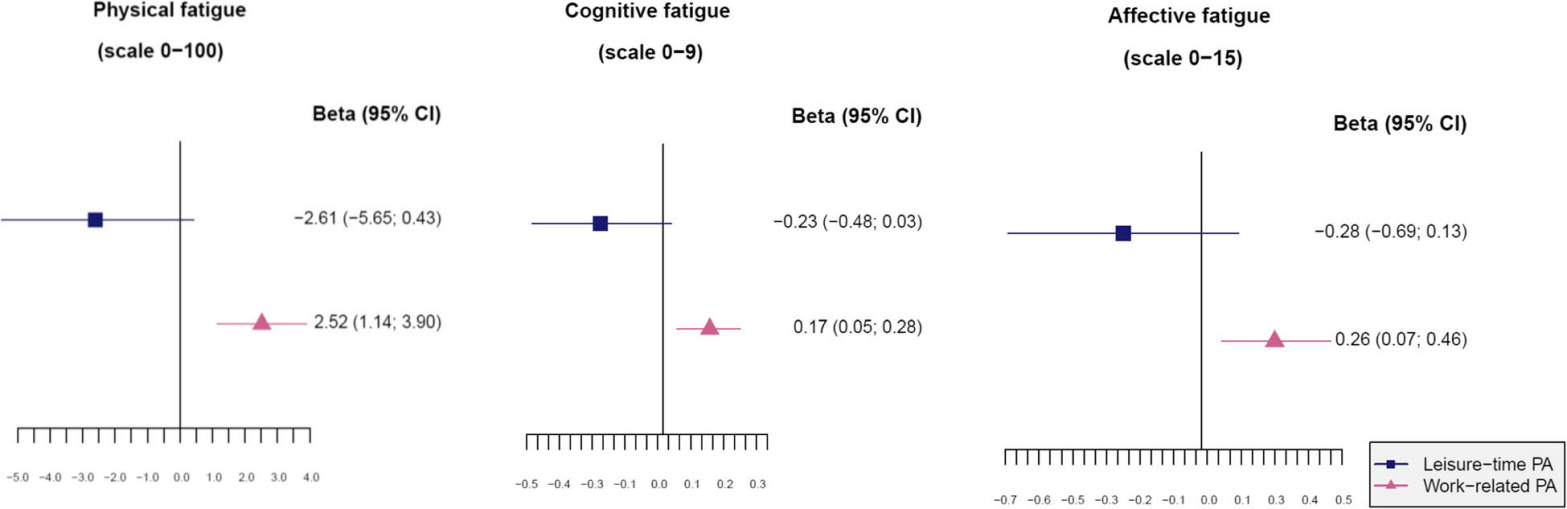
Associations between different domains of pre-diagnosis physical activity (MET hours per week in the last decade) and fatigue. Abbreviations: *CI* confidence interval, *PA* physical activity, *BMI* body mass index. Footnote: Linear regression analyses adjusted for age at baseline, sex, marital status, residential area, education, number of comorbidities at baseline, alcohol intake at baseline, smoking at baseline, BMI at baseline, cancer site, cancer stage, treatment, stoma, leisure time or work-related PA; leisure time PA including walking, cycling, sports; work-related PA including light work, hard work

**Fig. 4:**
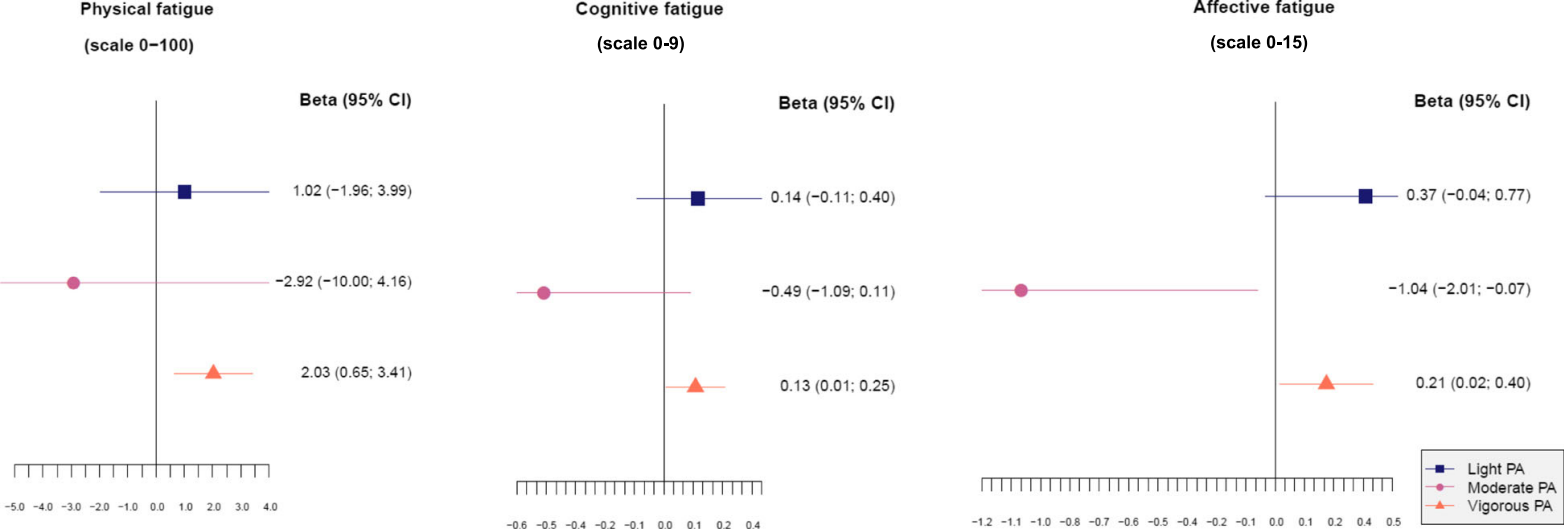
Associations between different intensities of pre-diagnosis physical activity (MET hours per week in the last decade) and fatigue. Abbreviations: *CI* confidence interval, *PA* physical activity, *BMI* body mass index. Footnote: Linear regression analyses adjusted for age at baseline, sex, marital status, residential area, education, number of comorbidities at baseline, alcohol intake at baseline, smoking at baseline, BMI at baseline, cancer site, cancer stage, treatment, stoma, light or moderate or vigorous PA; light PA including light work; moderate PA including walking; vigorous PA including hard work, cycling, sports

No association was found between a higher amount of pre-diagnosis leisure time PA (per 100 MET-h/wk) and any of the fatigue scales (Fig. 3). A higher amount of pre-diagnosis work-related PA (per 100 MET-h/wk) was significantly associated with higher physical, cognitive, and affective fatigue. No associations were found for pre-diagnosis light or moderate PA with fatigue, apart from a higher amount of pre-diagnosis moderate PA (per 100 MET-h/wk) being significantly associated with lower affective fatigue (Fig. 4). In contrast, a higher amount of pre-diagnosis vigorous PA (per 100 MET-h/wk) was significantly associated with higher physical, cognitive, and affective fatigue.

## Discussion

### Major findings

Higher levels of pre-diagnosis PA did not appear to be positively associated with fatigue among CRC survivors 5 years after diagnosis. Pre-diagnosis work-related PA and vigorous PA were even associated with higher physical, cognitive, and affective fatigue. In cross-sectional analyses, post-diagnosis PA was strongly associated with lower physical, cognitive, and affective fatigue. Moreover, survivors being physically active pre- and post-diagnosis and survivors who became physically active post-diagnosis scored significantly lower on all fatigue scales compared to survivors who remained inactive from pre- to post-diagnosis. The results of this study highlight the importance of ongoing PA throughout survivorship for the reduction of fatigue of CRC survivors, which is one of the most burdensome symptoms in cancer survivors [40]. However, the cross-sectional results should be interpreted with caution due to possible reverse causality.

### Relationship with previous findings

Our study found no beneficial effects of pre-diagnosis PA on long-term fatigue. This is in in line with a French study [19] which also used the EORTC QLQ-C30 fatigue subscale. That study, which included CRC survivors, also did not find an association between pre-diagnosis PA and fatigue in cancer patients 2 years after diagnosis. A possible explanation for these findings could be that in both studies, PA information was only available before diagnosis and 2 or 5 years after diagnosis. As such, it is not known how patients could have changed their PA habits over the course of their disease. Therefore it can be assumed that the time gap of five as well as 2 years might have been too long to still detect possible buffering effects [26–28] of pre-diagnosis PA and on fatigue two as well as 5 years post-diagnosis.

Of interest, we found that higher levels of pre-diagnosis work-related PA and vigorous PA were even positively associated with all fatigue scales. This suggests that survivors who had a physically demanding job before cancer diagnosis might still suffer from fatigue even years after their CRC diagnosis. Although all analyses within our study have been adjusted for education, the possibility of residual confounding, for example by lower socioeconomic status, has to be kept in mind. For example, CRC survivors who worked in manual labor might have lower autonomy, less pay, and more challenging working conditions (e.g. night shifts). These factors might be linked to depression and fatigue even years after diagnosis. Pertinent literature supports this assumption. It has been shown that cancer survivors with low education and low socioeconomic status were at higher risk for financial difficulties [41] and financial difficulties were associated with higher self-reported depression among cancer survivors [42]. However, the reported associations need to be interpreted with caution since effects were rather small using the 100 MET-h/wk classification and none of the differences were of clinical relevance.

The results regarding changes in PA support the cross-sectional findings on post-diagnosis PA and fatigue, and the assumption that ongoing PA may be important for fatigue of long-term CRC survivors. Only active maintainers and increasers had a significantly lower long-term fatigue compared to inactive maintainers, but no differences in fatigue were found for survivors decreasing their PA levels compared to those who stayed physically inactive. These findings may be explained by decreasers having a more severe health condition following CRC diagnosis and treatment which prevents them from maintaining PA levels compared to inactive maintainers who reported to be physically inactive pre- and post-diagnosis.

In line with our findings, several observational studies reported post-diagnosis PA to be associated with lower fatigue in CRC survivors [16–18, 43, 44]. However, a recent systematic review which performed a meta-analysis of randomized controlled trails, failed to show a significant association between PA and fatigue among CRC survivors, although in all studies PA was accompanied by reduced levels of fatigue [45]. Further, inconclusive results regarding the association between PA and fatigue for observational prospective studies were reported [45].

Although a multidimensional concept of fatigue is well accepted, most studies assessed the association between PA and physical fatigue unidimensionally. Therefore, studies might have missed some aspects of fatigue such as cognitive or affective fatigue and thus only few findings regarding the association of PA with multidimensional fatigue scales exist. Moreover, since some fatigue dimensions have been observed to behave differently it has been discussed that the different fatigue dimensions might not be expressions of one symptom but rather expressions of independent symptoms (multiple symptom concept) [46]. For example, some studies found physical fatigue to change in intensity during treatment or interventions that aim to reduce fatigue whereby mental fatigue did not change in intensity [47]. Also, specific subtypes of cancer-related fatigue with different correlates have been identified among long-term CRC survivors [48]. Therefore, it can be concluded that survivors might benefit from interventions targeted to the personal fatigue experience. For example, cancer survivors suffering from physical fatigue might benefit more from interventions that increase PA than survivors suffering from cognitive or affective fatigue for whom interventions such as mental training or psychosocial interventions might be more beneficial. Although the results of this study show that post-diagnosis PA was strongly associated with all fatigue scales, the association was lowest for PA and cognitive fatigue.

So far, most studies focused on fatigue shortly after CRC diagnosis. However, it has been reported that fatigue can persist years after diagnosis. Therefore, it is important to find out if PA is beneficial to mitigate long-term fatigue of CRC survivors. The findings of this study add to current knowledge that pre-diagnosis PA cannot replace ongoing PA after diagnosis among long-term CRC survivors, under the assumption that the association between ongoing PA and better fatigue is not entirely a result of reverse causality.

### Public health relevance

Fatigue is often reported as one of the most burdensome symptoms among cancer survivors [40] and it has been shown to affect QOL more than other symptoms such as pain or depression [8, 9]. Since fatigue can persist years into survivorship [49], it is of great relevance to find out more about possibilities that have the potential to decrease fatigue in CRC survivors, also in the long term. Contrary to our prior hypothesis, pre-diagnosis PA was not associated with lower fatigue and does not seem to protect CRC survivors against fatigue in the years after CRC diagnosis. Instead, ongoing PA after CRC diagnosis might be more important to mitigate fatigue among long-term CRC survivors and for survivors inactive at pre-diagnosis, it is never too late to start PA after diagnosis. Our findings might be used as a basis for more prospective studies and randomized controlled trials on the association between pre- and post-diagnosis PA and fatigue which might contribute to support specific PA interventions for CRC survivors.

### Strengths and limitations

Major strengths of our study include the analysis of a large population-based study sample, the prospective design, completeness of follow-up, comprehensive adjustment for confounders, and detailed investigations of differences in subgroups. Furthermore, results of the study are only based on long-term CRC survivors with a primary CRC diagnosis, and fatigue was assessed using validated and standardized questionnaires.

However, there are further limitations to consider. Firstly, due to the observational and partly cross-sectional study design, the results should be interpreted with caution because PA and fatigue may mutually affect one another and therefore our findings give only indirect support for recommendations of encouraging and maintaining PA after CRC diagnosis. Secondly, recall or desirability bias may have occurred through self-reported PA measurement at baseline and 5YFU. In addition, the PA questionnaires at baseline and at follow-up might not be directly comparable. Pre-diagnosis PA was assessed in a personal interview by trained interviewers asking for a wide range of different PA domains, whereby the short form of the validated IPAQ assesses less details about PA domains and was filled out by the survivors themselves. Resulting pre-diagnosis MET-h/wk were two to three times higher compared to MET-h/wk reported post-diagnosis and it cannot be determined whether and to what extent this difference can be attributed to differences in the assessment. Furthermore, MET-h/wk reported at baseline and at 5YFU were substantially higher than pertinent PA recommendations. To overcome this comparability issue, patients were grouped according to quantiles computed separately on the pre-diagnosis PA and post-diagnosis PA distribution instead of using PA recommendations. Furthermore, analyses on changes in PA were based on changes in the assessment specific quantiles instead of changes in MET-h/wk. Finally, residual confounding cannot be ruled out although adjustment for several potential confounders was performed.

## Conclusion

In conclusion, pre-diagnosis PA does not seem to be positively associated with fatigue among long-term CRC survivors. Instead our results support the need of ongoing PA after CRC diagnosis. However, due to the partly cross-sectional study design, these results should be interpreted cautiously. Randomized controlled trials are needed to provide information on the causality of the association between PA and fatigue among long-term CRC survivors and in turn could provide basis for individually-tailored PA recommendations to this population. Further prospective studies should focus on the association between PA and fatigue at multiple points in time pre- and post-diagnosis to determine if and how the effect of PA on fatigue changes.

## Supplementary information

**Additional file 1.**

## Data Availability

The datasets analysed during the current study are not publicly available due legal and ethical restrictions but are available from the corresponding author on reasonable request.

## Abbreviations

CRC: Colorectal cancer
QOL: Quality of life
PA: Physical activity
ICD-10: International Classification of Diseases, 10th Revision
5YFU: Five year follow-up
IPAQ: International Physical Activity Questionnaire
METs: Metabolic equivalent hours
MET–h/wk: Metabolic equivalent hours per week
Q: Quartile
FAQ: Fatigue Assessment Questionnaire
QLQ-C30: European Organization for Research and Treatment of Cancer QLQ-C30 questionnaire
BMI: Body mass index
ß: Beta values
CI: Confidence interval
