# Supplementary material for: Physical activity and long-term fatigue among colorectal cancer survivors – a population-based prospective study
Source: BMC Cancer. 2020 May 18;20:438. doi: 10.1186/s12885-020-06918-x (PMC7236466; doi:10.1186/s12885-020-06918-x)
Supplement: Supplementary file 1 — Additional file 1. [file 12885_2020_6918_MOESM1_ESM.docx]

| **Supplementary Table 1 Associations of pre-diagnosis physical activity (lifetime) and fatigue** | | | | | |  |
| --- | --- | --- | --- | --- | --- | --- |
|  | MET-h/wk | Q1  (<126.6) | Q2  (126.6-<188.6) | Q3  (188.6-<269.8) | Q4  (≥269.8) | |
| Total sample  N=1781 |  |  |  |  |  | |
| Fatigue Outcomes  (score range) |  | Mean  (95% CI) | Mean  (95% CI) | Mean  (95% CI) | Mean  (95% CI) | |
| Physical (0-100) |  | 41.0  (36.9; 45.1) | 39.3  (35.1; 43.4) | 42.6  (38.4; 46.9) | 46.0  (41.5; 50.4) | |
| Cognitive (0-9) |  | 2.2  (1.8; 2.5) | 2.2  (1.8; 2.5) | 2.4  (2.0; 2.8) | 2.4  (2.1; 2.8) | |
| Affective (0-15) |  | 3.8  (3.2; 4.4) | 3.9  (3.3; 4.4) | 4.5  (3.9; 5.0) | 4.5  (3.9; 5.1) | |
| **MET-h/wk:** metabolic equivalent hours per week; **Q**: physical activity quartile; **CI:** 95% confidence intervals; **BMI:** body mass index; Linear regression analyses adjusted for age at baseline, sex, marital status, residential area, education, number of comorbidities at baseline, alcohol intake at baseline, smoking at baseline, BMI at baseline, cancer site, cancer stage, treatment, stoma | | | | | | |
